# The transcription factor Uncx4.1 acts in a short window of midbrain dopaminergic neuron differentiation

**DOI:** 10.1186/1749-8104-7-39

**Published:** 2012-12-08

**Authors:** Tamara I Rabe, Gundula Griesel, Stephen Blanke, Andreas Kispert, Michael Leitges, Bert van der Zwaag, J Peter H Burbach, Frédérique Varoqueaux, Ahmed Mansouri

**Affiliations:** 1 Department of Molecular Cell Biology, Max Planck Institute of Biophysical Chemistry, Am Fassberg 11, Goettingen, 37077, Germany; 2 DFG Research Center for the Molecular Physiology of the Brain, CMPB, Humboldtallee 23, Goettingen, 37073, Germany; 3 Medizinische Hochschule Hannover, Institute of Molecular Biology, Carl Neuberg Strasse 1, Hannover, 30625, Germany; 4 Biotechnology Centre of Oslo, University of Oslo, Gaustadalleen 21, Oslo, 0349, Norway; 5 Rudolf Magnus Institute of Neuroscience, University Medical Center Utrecht, Department of Neuroscience and Pharmacology, Universiteitsweg 100, Utrecht, 3584 CG, The Netherlands; 6 Department of Molecular Neurobiology, Max Planck Institute for Experimental Medicine, Hermann-Rein-Strasse 3, Goettingen, 37075, Germany; 7 Department of Clinical Neurophysiology, University of Goettingen, Robert-Koch-Strasse 40, Göttingen, 37075, Germany; 8 Current affiliation: Department of Biomedical Genetics, University Medical Center Utrecht, Universiteitsweg 100, Utrecht, 3584 CG, The Netherlands

**Keywords:** Uncx4.1, Midbrain, mDA neurons, Ngn2, Pax6, Differentiation, Expression

## Abstract

**Background:**

The homeobox containing transcription factor Uncx4.1 is, amongst others, expressed in the mouse midbrain. The early expression of this transcription factor in the mouse, as well as in the chick midbrain, points to a conserved function of Uncx4.1, but so far a functional analysis in this brain territory is missing. The goal of the current study was to analyze in which midbrain neuronal subgroups Uncx4.1 is expressed and to examine whether this factor plays a role in the early development of these neuronal subgroups.

**Results:**

We have shown that Uncx4.1 is expressed in GABAergic, glutamatergic and dopaminergic neurons in the mouse midbrain. In midbrain dopaminergic (mDA) neurons Uncx4.1 expression is particularly high around E11.5 and strongly diminished already at E17.5. The analysis of knockout mice revealed that the loss of *Uncx4.1* is accompanied with a 25% decrease in the population of mDA neurons, as marked by tyrosine hydroxylase (TH), dopamine transporter (DAT), Pitx3 and *Ngn2.* In contrast, the number of glutamatergic Pax6-positive cells was augmented, while the GABAergic neuron population appears not affected in *Uncx4.1*-deficient embryos.

**Conclusion:**

We conclude that Uncx4.1 is implicated in the development of mDA neurons where it displays a unique temporal expression profile in the early postmitotic stage. Our data indicate that the mechanism underlying the role of Uncx4.1 in mDA development is likely related to differentiation processes in postmitotic stages, and where Ngn2 is engaged. Moreover, Uncx4.1 might play an important role during glutamatergic neuronal differentiation in the mouse midbrain.

## Background

Midbrain dopaminergic (mDA) neurons are organized in three different areas in the mammalian midbrain: the substantia nigra pars compacta (SNpc), the ventral tegmental area (VTA), and the retrorubal field (RRF). mDA neurons from the SNpc are associated with the nigrostriatal pathway while neurons from the VTA and RRF act in the mesolimbic pathway. The degeneration of neurons of the nigrostriatal pathway in Parkinson’s disease has attracted many researchers to identify and study the transcriptional network controlling mDA neuron development. Although several factors and determinants were found to be crucial for mDA neuron development like *Ngn2*[1,2], *Lmx1a/1b*[3], *Foxa1/2*[4,5], *Nurr1*[6], *Otx2*[7] and *ß-Catenin*[8,9] the molecular mechanisms controlling specification and differentiation of these neurons are not fully understood, and the knowledge of the transcriptional network is still incomplete.

Uncx4.1 is a homeobox containing transcription factor with 88% identity to Unc4 from *C.elegans*[10,11]. In mice, it is expressed in the caudal half of the somite, the developing kidney, and the central nervous system [10,11]. Functional analysis using knockout mice indicated that Uncx4.1 is required for the proper development of the axial skeleton [12,13]. In the central nervous system Uncx4.1 is detected in the spinal cord, the mesencephalon and the telencephalon [10,11]. Recent findings provided evidence for the involvement of Uncx4.1 in late development of the pituitary neural lobe [14], and the proliferation of neural progenitors, as well as neuronal survival in the mouse olfactory epithelium [15]. In contrast, the possible role of Uncx4.1 in the developing midbrain has not been addressed.

Here we show that in the ventral midbrain Uncx4.1 is found to localize with dopaminergic, glutamatergic and GABAergic postmitotic neurons at embryonic day (E) 11.5 of gestation. Molecular analysis of global loss-of-function mouse mutant revealed that mDA neurons are reduced in the absence of Uncx4.1, and this is corroborated by a partial downregulation of *Ngn2*. Interestingly the number of glutamatergic Pax6-expressing cells in the midbrain is increased at E13.5 while the Nkx6.1-positive neurons are not affected.

## Results

### *Uncx4.1* is expressed in the mantle layer of the developing midbrain

**Figure 1:**
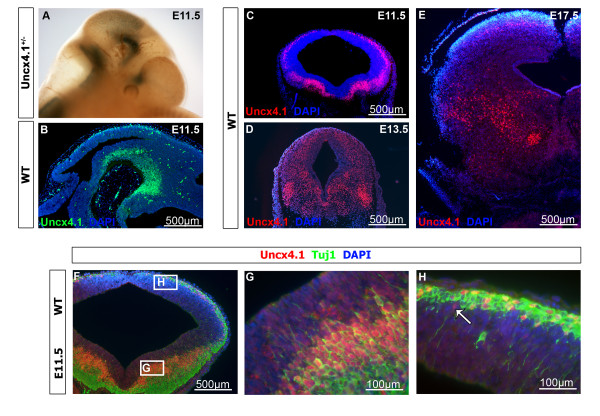
**The expression of Uncx4.1 in the developing mouse midbrain. (A)** X-gal staining of *Uncx4.1*^*+/− *^embryo at E11.5. **(B-E)** Immunohistochemistry (IHC) on coronal (D, C, E) and sagittal (B) sections of wild-type embryos showing the expression of Uncx4.1 in the developing midbrain at E11.5 (B-C), E13.5 (D) and E17.5 (E). (B, C) At E11.5 Uncx4.1 is expressed in the whole mantle layer of the embryonic midbrain. (D, E) At E13.5 and E17.5 Uncx4.1 expression appears in a salt-and-pepper pattern with Uncx4.1-negative cells. The strongest Uncx4.1 expression can be detected in the basal plate. **(F-H)** Double IHC with anti-Uncx4.1 and anti-Tuj1 on coronal sections of a wild-type embryo at E11.5. **(G, H)** Showing higher magnifications of F, referring to the labeling in the white box. The white arrow marks an Uncx4.1-positve, Tju1-negative cell. E, embryonic day.

**Figure 2:**
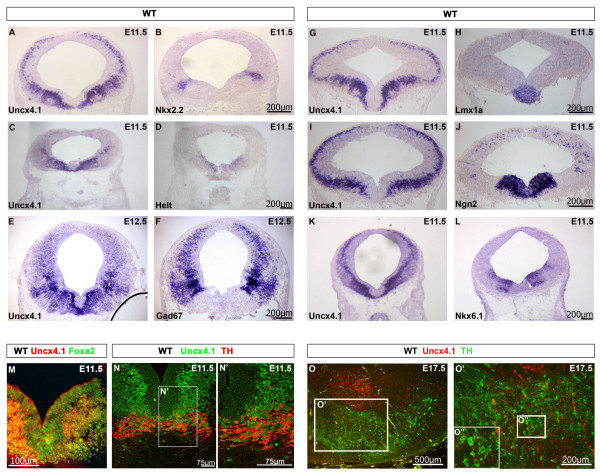
***Uncx4.1 *expression in embryonic mouse midbrain compared with expression of neuronal subtype markers. (A-L) ***In situ* hybridization analysis of *Uncx4.1* (A, C, E, G, I, K), *Nkx2.2* (B), *Helt* (D), *Gad67* (F), *Lmx1a* (H), *Ngn2* (J) and *Nkx6.1* (L) expression on coronal sections of E11.5 or E12.5 wild-type mouse midbrain. **(M, N)** Immunohistochemistry (IHC) on E11.5 wild-type sections reveals that Uncx4.1 is co-expressed with Foxa2 (M) and tyrosine hydroxylase (TH) (N) in postmitotic neurons of the ventral midbrain. **(N’)** Higher magnification of the inlay in N. **(O)** IHC on E17.5 wild-type sections shows that at E17.5 most of the TH-positive cells have lost Uncx4.1 expression. **(O’)** Higher magnification of the inlay in O. **(O”)** Higher magnification of the inlay in O’ showing that Uncx4.1 is not expressed in TH-positive cells at E17.5. E, embryonic day.

In the central nervous system *Uncx4.1* transcripts first appeared at E9.5 in the mesencephalon [10,13], but a detailed expression analysis of *Uncx4.1* in the developing mouse midbrain at subsequent stages so far has not been performed. At E10.5, transcripts were detected in the mantle layer of the basal and alar plate (data not shown). At E11.5 the signal in the basal and alar plate was increased and Uncx4.1 protein was detected in the ventral-most midbrain, close to the mesencephalic flexure (Figure 1B-C, Figure 2A and data not shown). At E13.5 *Uncx4.1* expression was scattered and the strongest labeling was observed in the lateral part of the basal plate (Figure 1D). At E17.5, Uncx4.1 expression remained scattered and the transcripts were distributed over the whole midbrain (Figure 1E). At all analyzed stages the expression of Uncx4.1 was similar throughout the rostral-caudal axis of the midbrain and it was excluded from the midbrain ventricular zone (VZ) , suggesting that it is confined to postmitotic cells.

Double immunohistochemistry (IHC) with Uncx4.1 and Tju1, a postmitotic neuron marker, revealed that nearly all Uncx4.1-positive cells in the mantle layer also express Tuj1 (Figure 1F-H). Only in the dorsal midbrain a few Uncx4.1-positive and Tju1-negative cells could be detected (Figure 1H). To examine which neuronal subtypes are marked by Uncx4.1 we compared the expression pattern of *Uncx4.1* and the neuronal subtype markers: *Lmx1a* (mDA neurons), *Gad67*, *Nkx2.2*, *Helt* (GABAergic neurons), *Nkx6.1* (glutamatergic neurons) and *Ngn2* (mDA and glutamatergic neurons). Shown in consecutive sections *Uncx4.1* expression domain appeared to co-localize at least partially with markers for most neuronal subgroups (Figure 2). This is true for *Gad67*, a general marker for GABAergic neurons in the mantle layer of the whole midbrain (Figure 2E-F), as well as for *Nkx2.2* (Figure 2A-B). As expected, *Helt*, which is expressed in the ventricular zone of GABAergic neurons [16,17], did not show overlapping expression with *Uncx4.1* (Figure 2C-D), confirming that *Uncx4.1* is absent from mesencephalic GABAergic progenitors. *Nkx6.1* labels a specific subset of glutamatergic neurons in the ventricular zone and the mantle layer [17,18]. The partially overlapping expression domains of *Nkx6.1* and *Uncx4.1* in the mantle layer indicate that *Uncx4.1* is expressed in midbrain glutamatergic neurons. In the most central area of the medial ventral midbrain region, where mature mDA neurons arise, *Uncx4.1* expression shared some expression domains with dopamine (DA) markers, namely *Lmx1a*[3] and *Ngn2*[1,2] (Figure 2G-H, I-J). In order to examine whether *Uncx4.1* is expressed in GABAergic, glutamatergic and DA neurons we performed IHC of Uncx4.1 together with tyrosine hydroxylase (TH) or Foxa2. The dopaminergic neuron marker *TH* started to be expressed around E11.5 in mature mDA neurons. At this stage, most TH-positive neurons expressed Uncx4.1 (Figure 2N), while this was not the case at E17.5 (Figure 2O, O’), where Uncx4.1 is downregulated in the VTA as well as SN. This finding suggests that *Uncx4.1* labels the majority of mature mDA neurons at early development, but is rapidly downregulated when matured. Co-localization of *Foxa2* and *Uncx4.1* was observed in the whole mantle layer within the expression domain of *Foxa2*, implying that *Uncx4.1* is expressed in mDA, GABAergic and glutamatergic neurons. Taken together, our data indicate that *Uncx4.1* expression is not confined to one neuronal lineage of mesencephalic neurons. Rather, Uncx4.1 is expressed in most subtypes of midbrain neurons, but is excluded from their proliferating progenitors. These data suggest that *Uncx4.1* is engaged in processes of postmitotic differentiation.

### Loss of *Uncx4.1* causes reduction of mDA neurons in the ventral midbrain

**Figure 3:**
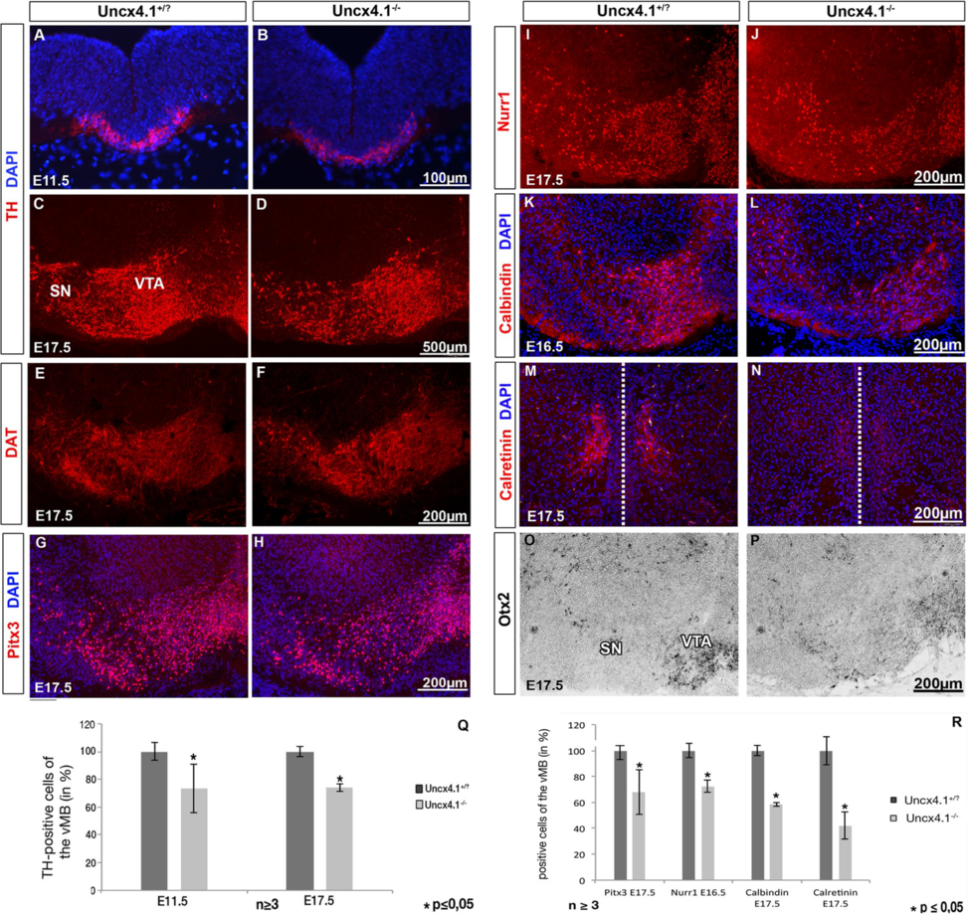
**Midbrain dopaminergic neurons are reduced in *Uncx4.1***^***−/− ***^**embryos. (A-D)** Compared with control, tyrosine hydroxylase (TH)-positive neurons are reduced in *Uncx4.1*^*−/− *^ embryos at E11.5 (A-B) and E17.5 (C-D). **(E-N)** Immunohistochemistry (IHC) reveals a reduced expression of dopamine transporter (DAT) (E-F), Pitx3 (G-H), Nurr1 (I-J) Calbindin (K-L) and Calretinin (M-N) in *Uncx4.1*^*−/−*^ embryos compared to control. **(O-P)***In situ* hybridization (ISH) using a probe against Otx2 shows that the expression of Otx2 is reduced in *Uncx4.1*-deficient mice compared to control. **(Q, R)** Quantification of TH-, Pitx3-, Calbindin- and Calretinin-positive cell numbers in the ventral midbrain of *Uncx4.1*-deficient and control embryos. Error bars represent the standard deviation. *P* values <0.05 were assessed as statistically significant. E, embryonic day; SN, substantia nigra; vMB: ventral midbrain, VTA, ventral tegmental area.

To examine the role of *Uncx4.1* in mDA development, we analyzed the expression of the dopamine transporter (DAT) and TH in *Uncx4.1-*deficient embryos at E11.5 and E17.5. We observed a clear reduction of DAT expression at E17.5 (Figure 3E-F). Therefore, the number of mDA neurons was assessed. Although the distribution of TH-positive cells was normal in control and *Uncx4.1* mutant embryos, the number of TH-positive cells was found significantly reduced (Figure 3A-D, Q). Interestingly, the decrease of TH-positive cells at E11.5 was similar to the reduction observed at E17.5 (both −26%, Figure 3Q). The partial loss of mDA neurons was further confirmed by IHC for Pitx3, Nurr1, Calretinin and Calbindin. Pitx3 specifically labels mDA neurons [19-22] and the reduction of Pitx3-positive cells (−31%), correlated with the diminution of TH-positive cells (Figure 3I-J, R). A similar observation was made for the Nurr1-labeled cells at E16.5 (reduction of 24%). The number of Calbindin- and Calretinin-marked cells, which are both expressed in a subset of mDA neurons [23,24], was drastically decreased in *Uncx4.1*^*−/−*^ embryos (−43% and −58%, respectively, Figure 3M-P, R). The early onset in the reduction of TH-positive cells in the ventral midbrain and the corresponding downregulation of all tested markers indicates that *Uncx4.1* plays an early role during development of mDA neurons. Furthermore, the dramatic reduction of Calbindin in the ventral midbrain suggests that the mDA neurons of the VTA are more affected as compared to the SN, since Calbindin is preferentially expressed by VTA neurons [24]. To confirm the findings that the mDA neurons of the VTA are strongly affected in the absence of Uncx4.1, we analyzed the expression of *Otx2* at E17.5 in *Uncx4.1-*deficient embryos. Otx2 is a transcription factor, which selectively labels postmitotic neurons of the VTA but not of the SN at E17.5, and it is expressed in a subset of Calbindin-positive neurons in this brain area [25]. Accordingly, *Otx2* was downregulated in the VTA, confirming the findings from the Calbindin and Calretinin staining (Figure 3O-P). The partial depletion of mDA neurons may be a consequence of impaired cell proliferation or survival defects. To examine both possibilities we used 5-bromo-2'-deoxyuridine (BrdU) labeling for proliferation analysis and the terminal deoxynucleotidyl transferase dUTP nick end labeling (TUNEL) assay for investigating apoptosis. At E11.5, 24 hours after BrdU injection, as well as at E14.5, 45 minutes following BrdU injection, no alterations in numbers of BrdU-positive cells were observed (Additional file 1: Figure S1 A-B and data not shown). To further confirm that the decreased number of mDA neurons is not caused by alterations in cell proliferation of this neuronal subgroup, we analyzed the BrdU-labeled cells together with TH at E11.5, 24 hours following BrdU-injection. In the wild-type (WT) as well as in mutant embryos, we could not detect any difference in cell proliferation (Additional file 1: Figure S1 C-D). Apoptotic cells were also not detected at E11.5, and E17.5 (data not shown). We concluded that defects in cell proliferation or apoptosis are not involved in the DA neuron loss provoked by Uncx4.1 depletion.

### The expression of *Ngn2* is downregulated in *Uncx4.1-*deficient embryos

**Figure 4:**
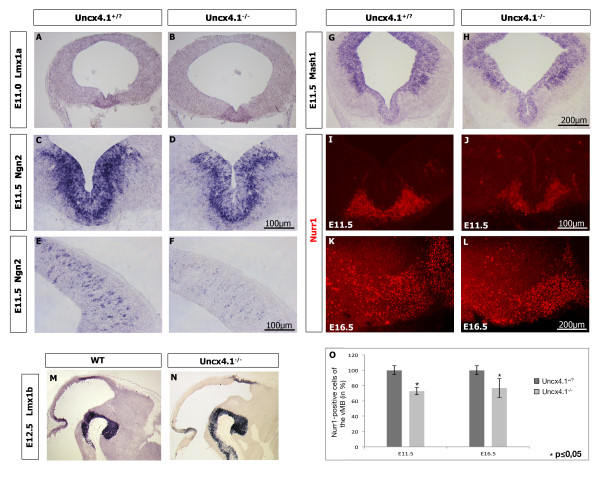
**Analysis of early mDA markers in *Uncx4.1*-deficient embryos showed that Ngn2-expression is reduced in mutant embryos.** A detailed analysis of Nurr1 expression in early and late embryonic stages of *Uncx4.1*^*−/− *^embryos revealed that the loss of Nurr1 is consistent during midbrain dopaminergic neurons (mDA) development. **(A-B)***In situ* hybridization (ISH) using *Lmx1a* reveals no difference in the expression of *Lmx1a* in *Uncx4.1*^*−/−*^ embryos at E11.0. **(C-F)** Reduction of *Ngn2* in the ventral (C-D) and dorsal (E-F) midbrain of *Uncx4.1*^*−/−*^ embryos compared to control using ISH. **(G-H)** At E11.5 *Mash1* expression is normal in *Uncx4.1*-deficient embryos compared to control. **(I-L)** Showing Nurr1 expression in control and *Uncx4.1*^*−/−*^ embryos by immunohistochemistry (IHC). **(O)** Nurr1 is significantly reduced at E11.5 and E16.5 compared to wild-type (WT) animals. Error bars represent the standard deviation. *P* values <0.05 were assessed as statistically significant. At least three animals per stage and genotype were analyzed. **(M, N)** ISH using *Lmx1b* reveals no difference in *Lmx1b* expression in *Uncx4.1*^*−/−*^ embryos. E, embryonic day; vMB, ventral midbrain.

**Figure 5:**
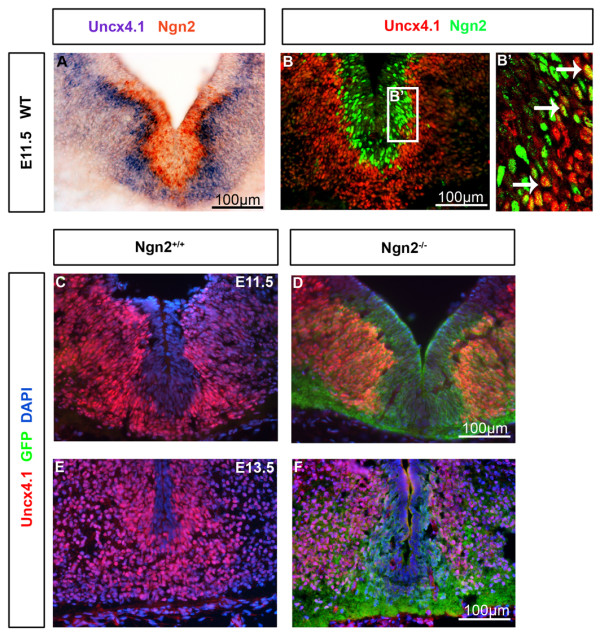
**Uncx4.1 and Ngn2 are co-expressed in some immature neurons in WT embryos, whereas Uncx4.1 is missing in the ventral midline of *Ngn2***^***−/− ***^**embryos. (A-B)** Analyzes of the expression of Uncx4.1 and Ngn2 using *in situ* hybridization ISH (A) and immunohistochemistry (IHC) (B) revealed that Uncx4.1 and neurogenin 2 (Ngn2) are co-expressed in some immature neurons of the ventral midbrain. It appears that on the mRNA level more double-positive cells are detected, as compared to IHC staining. **(B’)** display confocal microscopy higher magnification of the inlay in B. **(C-F)** IHC against Uncx4.1 and green fluorescent protein (GFP) on coronal sections of E11.5 (C-D) and E13.5 (E-F) embryos, showing that Uncx4.1 is depleted at the midline in the ventral midbrain of *Ngn2*^*−/−*^ embryos as compared to WT. E, embryonic day; WT, wild-type.

Since it appeared that mDA neuron reduction was not caused by impaired proliferation or cell death, we next investigated which factors may be affected by the absence of *Uncx4.1,* provoking the partial depletion of mDA neurons. Since *Lmx1a, Lmx1b*, and *Ngn2* are involved for proper establishment of mDA neurons [1-3,26,27], we analyzed their expression in E11.0 (*Lmx1a*), E11.5 (*Ngn2*) or E12.5 (Lmx1a and *Lmx1b*) *Uncx4.1*^*−/−*^ embryos. As expected, *Lmx1a* and *Lmx1b* expression was normal in *Uncx4.1-*deficient embryos (Figure 4A-B, M-N and data not shown) but surprisingly the level of *Ngn2* mRNA was reduced (Figure 4, C-F). Interestingly, the downregulation of *Ngn2* transcripts was also observed in the dorsal part of the midbrain (Figure 4, E-F). Nurr1 expression was found nearly lacking in *Ngn2* mutant mice at E11.5, but Nurr1-positve cells were rescued at later stages of development [1]. We therefore investigated the expression of *Nurr1* in the developing *Uncx4.1* mutants and observed at E11.5 a 28% loss of Nurr1-positive cells compared to control embryos (Figure 4, I-J, O). A similar reduction of 23% occurred in E16.5 embryos (Figure 4, K-L, O). It has been previously speculated that the rescue of Nurr1-positive cells in *Ngn2*^*−/−*^ embryos is due to a compensation by the proneuronal gene *Mash1*[1]. Its expression was completely missing at the ventral midline in E11.5 *Ngn2*^*−/−*^ embryos and was recovered at E13.5 [1]. In *Uncx4.1*-deficient midbrain the expression of *Mash1* was found to be normal at E11.5 (Figure 4, G-H), indicating that unlike in *Ngn2*^*−/−*^ embryos, Nurr1-positive cells do not recover in the absence of *Uncx4.1*. Since neurogenin 2 (Ngn2) is required for the proper generation of mDA neurons, these results indicate that the alteration of *Ngn2* expression may be responsible for mDA neuron reduction in the absence of Uncx4.1. We therefore analyzed the expression of Uncx4.1 and Ngn2 in more detail. Ngn2 starts to be expressed in mDA progenitors and immature mDA neurons around E10.75 [1]. At E11.5 Ngn2 and Uncx4.1 co-positive cells were detected in the mantle layer of the ventral midbrain (Figure 5A-B, B’). It appears that on the mRNA level more double-positive cells were detected. The expression of *Unxc4.1* in the ventral midbrain of *Ngn2*^*−/−*^ embryos was absent in the most ventral part of the midbrain at E11.5 and E13.5 (Figure 5C-F), where mDA neurons are located. This is consistent with the finding that Ngn2 is needed for the generation of postmitotic mDA precursors and that the progenitors are arrested in the absence of Ngn2 at E11.5 [1]. In contrast, *Uncx4.1* expression in dorsal midbrain was not affected (data not shown). To further analyze whether Uncx4.1 and Ngn2 interact with each other, we performed a co-immunoprecipitation assay. Our results show that there is no interaction between Ngn2 and Uncx4.1 (Additional file 2: Figure S2), suggesting that these proteins do not undergo such a protein-protein interaction. Uncx4.1 may still interact with Ngn2 promoter. However, the palindromic putative binding site for Uncx4.1 (TAATYNRATTA) could not be identified on the Ngn2 locus. Taken together our findings suggest that the partial depletion of mDA neurons in *Uncx4.1*-deficient embryos may be a consequence of *Ngn2* downregulation during an early phase of mDA neuron differentiation. How Uncx4.1 regulates *Ngn2* expression remains elusive. It is possible that the observed downregulation of *Ngn2* expression is mediated by an unknown factor that interacts with Uncx4.1.

### Normal development of midbrain GABAergic neurons in the absence of *Uncx4.1*

**Figure 6:**
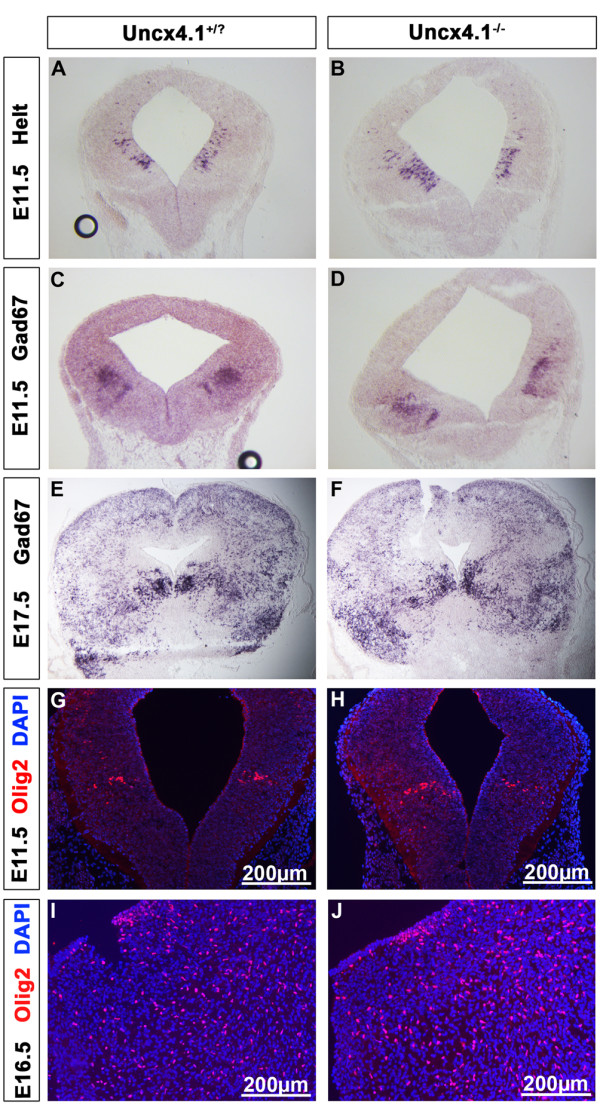
**Midbrain GABAergic neurons are not affected in *Uncx4.1-*deficient embryos. (A-B)***In situ* hybridization (ISH) of *Helt* on E11.5 coronal sections reveals no difference in the absence of *Uncx4.1*. **(C-F)** The GABAergic marker *GAD67* is expressed normally in *Uncx4.1*^*−/− *^embryos at E11.5 (C-D) and E17.5 (E-F) compared to control animals. **(G-J)** Immunohistochemistry (IHC) against Olig2 shows no difference in control and *Uncx4.1*^*−/− *^embryos at E11.5 **(G-H)** and E16.5 **(I-J)**. E, embryonic day.

Our analysis indicated that the loss of *Uncx4.1* is accompanied by downregulation of *Ngn2* expression, resulting in a partial depletion of mDA, without affecting cell proliferation or cell death. We therefore asked the question whether a cell identity switch might have occurred in the *Uncx4.1-*deficient midbrain and if these cells now ectopically express GABAergic neuron markers. Kala *et al*. showed that GABAergic neurons are located in the VTA-SNpr in late development [28]. To investigate whether mDA neurons switch their neuronal identity into a GABAergic destiny in the absence of *Uncx4.1*, or whether ectopic expression of GABAergic markers occurs in the mDA domain*,* we studied the expression of *Helt* and *Mash1* which are essential for development of midbrain GABAergic neurons [17,29,30]. The expression of *Helt* (Figure 6, A-B) and *Mash1* (Figure 4, G-H) was unaltered in the absence of *Uncx4.1*. To exclude the possibility that *Uncx4.1* influences GABAergic neuron development postmitotically, we analyzed expression of *Gad67* and *Olig2* in *Uncx4.1-*deficient embryos. While *Olig2* is confined to a specific subgroup of GABAergic neurons in the mantle layer *Gad67* is found in all these neurons [17,28]. We did not observe a difference in the expression of *Gad67* or Olig2 at early and late developmental stages (Figure 6, C-J). Together, these data demonstrated that inactivation of *Uncx4.1* does not result in the ectopic activation of GABAergic neuron markers in the mDA domain. Therefore, we next examined glutamatergic neuron development in *Uncx4.1*-deficient embryos.

### Increased numbers of Pax6-positive glutamatergic neurons in the ventral midbrain upon *Uncx4.1* deficiency

**Figure 7:**
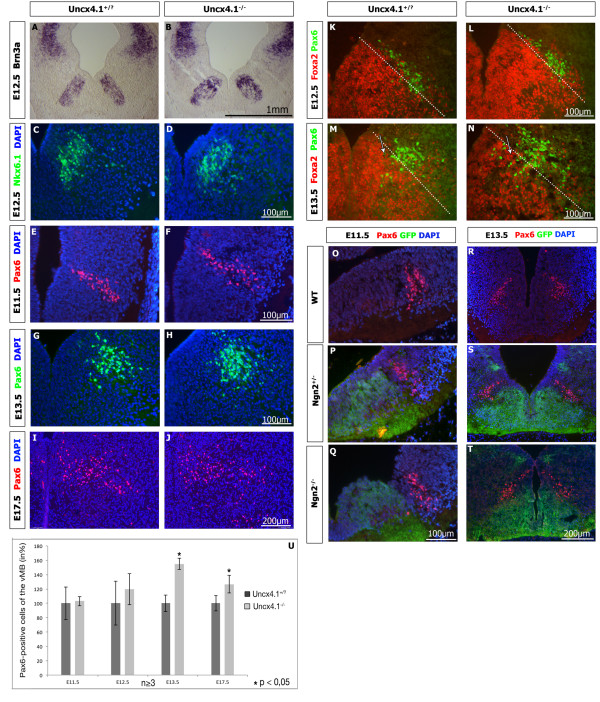
**Analysis of midbrain glutamatergic markers. (A-B)***Brn3a* expression is increased in the ventral midbrain of *Uncx4.1*^*−/−*^ embryos compared to controls. **(C-D)***Nkx6.1* expression is normal in E12.5 embryos, indicating a proper establishment of glutamatergic progenitors. **(E-F)** and **(K-L)** The numbers of Pax6-expressing cells are normal at E11.5 and E12.5 in *Uncx4.1*-deficient embryos compared to control. **(G-J)** and **(M-N)** Immunohistochemistry (IHC) reveals increased numbers of Pax6-expressing cells at E13.5 and E17.5 in the absence of *Uncx4.1*. **(U)** Quantification of Pax6-expressing cells in the ventral midbrain of *Uncx4.1*^*−/−*^ and control embryos. Error bars represent the standard deviation. *P* values <0.05 were assessed as statistically significant. (M-N) Co-labeling with Foxa2 revealed that Pax6 cells are extended more ventrally at E13.5 (white arrows). The dashed line marks the border in the Foxa2 expression domain. **(O-T)** Pax6 and green fluorescent protein (GFP) staining of *Ngn2*^*−/−*^ embryos shows no difference in the numbers of Pax6-expressing cells in the basal plate of the ventral midbrain at E11.5 (O-Q) and E13.5 (R-T). E, embryonic day; vMB: ventral midbrain.

Glutamatergic neurons are located in the embryonic dorsal midbrain as well as in the ventral midbrain, and adjacent to the mDA neuron domain [17,28,31]. *Brn3a* was used as a marker for glutamatergic red nucleus neurons [32] that are located adjacent to the mDA neuron domain [17,28,33]. Using *in situ* hybridization (ISH) we observed an upregulation in the expression of *Brn3a* in the ventral but not in the dorsal midbrain at all stages analyzed from E11.5 to E13.5 (Figure 7, A-B, and data not shown). Recently, it was reported that Nkx6.1 plays a crucial role for proper development of Brn3a-positive red nucleus neurons [18]. Applying IHC no apparent difference in Nkx6.1 expression at E12.5 (Figure 7 C-D) or E13.5 (data not shown) was detected. Interestingly, all Pax6-positive cells of the ventral midbrain are glutamatergic [28]. Using IHC Pax6 expression was not affected at E11.5, and E12.5 in mutant embryos (Figure 7E-F, U). However, starting with E13.5 the number of Pax6-expressing cells was increased by 55% (Figure 7G-H, U) and by around 26% at E17.5 (Figure 7I-J, U). Double IHC against Pax6 and Foxa2 revealed, unlike at E12.5, that the Pax6 expression domain was slightly extended ventrally at E13.5 (Figure 7K-N). Further analysis using ISH and IHC indicated that the expression of *Uncx4.1* is not affected in the midbrain of *Pax6-*deficient embryos at E11.5 and E17.5 (data not shown). We then asked whether the observed upregulation of Pax6 expression in *Uncx4.1-*deficient midbrain is caused by the diminution of *Ngn2* transcription in *Uncx4.1* mutant embryos. However, Pax6 expression was not modified in the basal plate of *Ngn2*^*−/−*^ embryos at all analyzed stages, indicating that the increase of Pax6-positive cells in *Uncx4.1*^*−/−*^ embryos may not be directly related to the reduction of *Ngn2* (Figure 7O-T). In none of the above realized experiments we noted ectopic expression of glutamatergic markers in the mDA domain, indicating that a fate switch to a glutamatergic identity had not occurred. Our findings demonstrate that *Uncx4.1* may play a critical role in the development of glutamatergic neuron subgroups in the ventral midbrain, and that the increased numbers of Pax6-positive neurons are not directly related to the partial depletion of *Ngn2*.

### Conditional inactivation of *Uncx4.1* leads to a partial loss of mDA neurons in the SN of adult mutant mice

**Figure 8:**
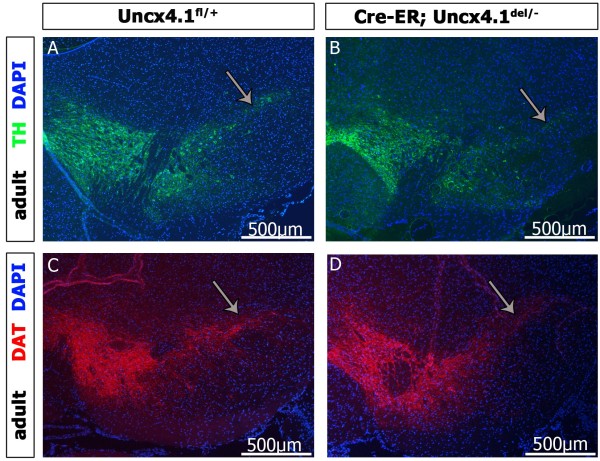
**Conditional inactivation of *Uncx4.1 *at E12.5 leads to defects in the mDA neuron domain. (A-B)** Tyrosine hydroxylase (TH) staining on midbrain sections of Uncx4.1^fl/+^ and Cre-ER:Uncx4.1^del/-^ mice. **(C-D)** Immunohistochemistry (IHC) of dopamine transporter (DAT) on ventral midbrain sections of *Uncx4.1*^*fl/+*^ and Cre-ER:*Uncx4.1 *^*del/- *^mice. Grey arrows show the substantia nigra (SN).

Since *Uncx4.1-*deficient mice die shortly after birth [12,13], we created a conditional *Uncx4.1* mutant in order to examine mDA neurons in the adult midbrain. This conditional Cre-ER:*Uncx4.1* mutant was obtained by crossing the floxed *Uncx4.1* knockout line with an inducible Cre-ER mouse line [34] and injected tamoxifen at E12.5. Cre recombination efficiency was analyzed on P0 brains using an Uncx4.1 antibody. Around 90% of the Uncx4.1 signal was lost in Cre-ER:*Uncx4.1*^*del/-*^ animals. Under such tamoxifen application conditions, and as reported for the Cre-ER [34], we assume that at E14.5, 90% of Uncx4.1 gene activity is lost. To analyze mDA neurons in adult Cre-ER:*Uncx4.1*^*del/-*^ mice we performed IHC against TH and DAT. Such mutants exhibited a weak, but statistically not significant reduction in TH-positive cells compared to control animals (n = 3). However, the most lateral neurons of the SNpc always appeared reduced in mutant as compared to control midbrain (Figure 8, A-D, grey arrows and Additional file 3: Figure S3). Since almost all mDA precursors have been generated before E12.5 when tamoxifen was injected, but differentiation into mature neurons and migration still takes place, our findings suggest that Uncx4.1 might be involved in maturation and/or cell migration, but not in the generation of mDA neurons.

## Discussion

In recent years enormous efforts were made to identify and characterize transcription factors controlling mDA neurogenesis, including Msx1 [3], Foxa2 [4], Lmx1a [3], Ngn2 [1,2], Nurr1 [6,35], En1/2 [36], Lmx1b [37] and Pitx3 [19,21,38,39]. In the transition from VZ mDA progenitors via precursors to mature mDA neurons, these factors provide a complex regulatory network required for full expansion of the heterogeneous mDAergic field of the midbrain [40]. In this study we provide evidence for an important novel role of the transcription factor Uncx4.1 in mDA neuron development. This role is exerted and confined in the critical phase of transition from progenitors to mature mDA neurons, roughly between E9.0 to E12.5, and it engages the proneural gene *Ngn2*. During this temporal window *Uncx4.1* is required to sustain the expression of *Ngn2*. This suggests that Uncx4.1 is mainly a differentiation factor that may affect essential properties of mDA neurons in this period. For instance, our analysis suggests a possible implication of Uncx4.1 in the migration process of mDA precursors. In this respect it is important to note that *Uncx4.1* deficiency causes a connectivity defect in the pituitary complex [14]. In this study we found that hypothalamic magnocellular neurons, neuroendocrine cells in the hypothalamus, fail to fully innervate the posterior pituitary, and instead run through the anterior pituitary. This points to an axon guidance defect in this neuronal system.

The current study also demonstrates that midbrain expression of Uncx4.1 is not exclusive for mDA neurons. It also includes GABAergic and glutamatergic neurons. We show that the loss of *Uncx4.1* provokes an increase in the numbers of glutamatergic Pax6-marked cells in the ventral midbrain, suggesting an additional important function for *Uncx4.1* activity in glutamatergic neurogenesis.

### The expression of *Uncx4.1* in mDA neurons

Developing midbrain dopaminergic neurons can be subdivided into three different groups. They arise from proliferating committed neural stem cells, first as postmitotic progenitors, then postmitotic precursors, and finally mature mDA neurons. Mature mDA neurons express the enzyme TH that is involved in the dopamine biosynthesis. In this study we could show that *Uncx4.1* is expressed in nearly all TH-positive cells at E11.5 indicating that it is present in mature mDA neurons. *Uncx4.1* expression seems to be induced in mDA progenitors that express *Ngn2*[1,2]. This timing resembles the expression of Nurr1, a central transcription factor in the cell-specific expression and regulation of genes in mDA neurons [41]. In the whole midbrain, Uncx4.1 is absent from the VZ, but expressed in postmitotic neurons. Using IHC and ISH, we further demonstrate that Uncx4.1 is not restricted to a specific lineage but that it is present in most neuronal subgroups during midbrain development. This finding is consistent with a recent report demonstrating *Uncx4.1* expression in all neuronal lineages of the olfactory bulb [15]. In conclusion, our findings support the idea that this transcription factor might have a general and cell-type independent role during neuronal development [15]. Furthermore, it may also promote particular properties of specific neuronal type, such as mDA neurons.

### The potential role of *Uncx4.1* in mDA neuron development

Our analysis of *Uncx4.1*^*−/−*^ embryos revealed a reduced expression of several mDA neuron markers, and including TH, DAT, Nurr1, and Pitx3. In addition, the numbers of Calbindin- and Calretinin-marked cells were severely decreased in this context. Calbindin and Calretinin are preferentially expressed in VTA neurons [23,24] whereas Calbindin labels mDA neurons of the VTA [2,42]. Therefore, the more pronounced reduction of Calbindin- and Calretinin-marked neurons indicates, that VTA neurons are possibly more affected by the absence of *Uncx4.1* than those of the SN. This finding was further corroborated by the reduced expression of *Otx2*, a transcription factor expressed in mature mDA neurons of the VTA but not of the SN at E17.5 embryos and adult mice [25]. Although our findings indicate that the mDA neurons of the VTA are more affected than those of the SN, we could also observe reduced numbers of mDA neurons in the SN. However, the minor decrease of mDA neurons in *Uncx4.1*-deficient mice is consistent with the notion that this factor only partially affects the differentiation of mDA neurons. Moreover, it appears that, although decreased, the level of *Ngn2* expression is still sufficient for the generation DA neurons. It is also reasonable to assume that the loss of Uncx4.1 is compensated by another factor. It was shown in *C. elegans* that Unc-4, a homolog of Uncx4.1 [10,43], acts together with a co-repressor and that these proteins are involved in the correct specification of the transmitter phenotype [22]. The persistent lower numbers of TH-positive neurons in *Uncx4.1* mutants at E11.5, as well as at E17.5 of development, are consistent with the idea that the absence of *Uncx4.1* does not provoke a delay in mDA neuron generation. In contrast to what has been described for the olfactory bulb of *Uncx4.1*^*−/−*^ mice, where cell death was reported [15], the lack of *Uncx4.1* in the midbrain is not accompanied by an alteration in proliferation or apoptosis. This may suggest that in *Uncx4.1*-deficient mice the loss of a subpopulation of dopaminergic neurons is compensated by the accumulation of another neuronal cell type, which will be discussed below. Since *Uncx4.1* is not expressed in the VZ where cellular specification occurs, and in addition, the majority of Uncx4.1-positive cells in the ventral midbrain are co-positive for TH at E11.5, it is more likely that Uncx4.1 acts during differentiation and not during specification of mDA neurons. The absence of Uncx4.1 from TH-positive cells at later stages (E17.5) supports the idea that Uncx4.1 is primarily acting during a short, critical window of differentiation of mDA neurons. Accordingly, the numbers of mDA neurons is unaffected at adult stages when *Uncx4.1* was conditionally inactivated at E12.5, although it seems that the neurons of the SN are reduced. After their generation, mDA neurons first migrate along the dorsal ventral axis before they migrate laterally to their final destination. Since we could not recognize a difference in the cell content of mDA neurons in the conditional mutant mice, after deletion of Uncx4.1 at E12.5, but a reduction in the SN, this may suggest that Uncx4.1 is involved in the proper migration of these neurons. This is consistent with an earlier finding, where we could show that hypothalamic magnocellular neurons fail to fully innervate the posterior pituitary, and instead they connect to the anterior pituitary [14]. Moreover, it was shown that Unc-4 is involved in the correct establishment of the innervation of VB motor neurons in *C.elegans*[44-46]. This favors the hypothesis that Uncx4.1 is possibly implicated in the correct axonal guidance of mDA neurons and that the migration of mDA neurons to the SN is altered. The proneural gene *Ngn2* is required for neuronal differentiation of mDA progenitors and starts to be expressed around E10.75 [1,2]. Within the mDA neuron population Ngn2 is exclusively present in dopaminergic neuron progenitors and immature neurons. In the absence of Uncx4.1, *Ngn2* expression is down-regulated, providing additional evidence that the observed alterations in mDA neurons in *Uncx4.1* mutant may be related to a differentiation defect in the progenitor or immature neuron pool. The transcription factor Nurr1 is expressed in immature and mature neurons and found essential for their maintenance [6,47,48]. It has been shown that Nurr1-positive cells are nearly absent in *Ngn2*-deficient embryos at E11.5 and that they recover around E13.5 [1]. In contrast to *Ngn2* mutants, we did not observe such a recovery of Nurr1-positive cells in *Uncx4.1*-deficient mice. This is possibly due to the unaltered expression of *Mash1* in the ventral midbrain [1]. Interestingly, we could not detect Uncx4.1 expression in the ventral midline of *Ngn2*-deficient midbrain. However, this may simply be explained by the nonestablished postmitotic neurons within the mDA domain of *Ngn2* mutant at the analyzed time points. The Co-IP experiment and the fact that no binding sites for Uncx4.1 were identified on the Ngn2 locus provide evidence that Uncx4.1 and Ngn2 do not interact with each other. Therefore it is reasonable to assume that another unknown factor may be involved in the partial loss of mDA neurons provoked in *Uncx4.1* mutant embryos, and that the downregulation of *Ngn2* is indirectly related to the moderate decrease of this neuron population. Overall, our results point to a mechanism of Uncx4-mediated differentiation events in the critical E9.0 to 12.5 time window, and indirectly engaging *Ngn2*.

### The potential role of *Uncx4.1* in midbrain glutamatergic neuron development

As mentioned above the loss of a subpopulation of mDA neurons may be compensated by the accumulation of another neuronal cell type. In fact, we could not detect ectopic expression of glutamatergic neuron markers in the mDA domain, which makes it unlikely that mDA neurons change their cell fate. However, we found increased *Brn3a*-mRNA signal in the ventral midbrain leading to the idea that Uncx4.1 acts on glutamatergic neuron development. The increase in the numbers of Pax6-positive neurons at E13.5 in *Uncx4.1*-deficient mice favors this hypothesis. The transcription factor Brn3a is expressed in glutamatergic red nucleus neurons, which are located adjacent to the mDA domain at E11.5 [17,28,31,32,49,50]. Since Nkx6.1 is required for the proper development of Brn3a-positive red nucleus neurons [18], the unaltered numbers of Nkx6.1-positive cells indicate that the ventral glutamatergic progenitors are normally established. During development, the transcription factor Pax6 is expressed in a subset of glutamatergic neurons [28]. In this study, we demonstrate that the number of Pax6-expressing cells is increased in *Uncx4.1*^*−/−*^ embryos starting with E13.5 and this alteration persists until at least E17.5. The increase in Pax6-positive cell number is not related to the downregulation of *Ngn2* transcription. This is corroborated by the nonaltered expression of Pax6 in *Ngn2*^*−/−*^ mutant mice. Taken together, our findings suggest that Uncx4.1 acts on glutamatergic neuron differentiation. In addition, the normal expression of *Uncx4.1* in *Pax6*-deficient midbrain at E11.5 may indicate that Uncx4.1 acts upstream of *Pax6*. Interestingly *Ngn2*-labeled glutamatergic neurons are more reduced in the dorsal than in the ventral midbrain of *Uncx4.1*^*−/−*^ embryos. In this context, it was shown that Ngn2 promotes the glutamatergic neurotransmitter fate by repressing the GABAergic cell fate in the mouse cortex [51]. However, we did not observe any difference in GABAergic marker expression in *Uncx4.1*-deficient mice, suggesting that the remaining low level of *Ngn2* is still sufficient to mediate glutamatergic over GABAergic neuron development. This finding is supported by the unaltered expression of the glutamatergic neuron marker *Brn3a* in the dorsal midbrain. Another explanation for the normal expression of GABAergic markers might be that *Ngn1* compensate for the loss of *Ngn2.* Alternatively, *Ngn2* itself may not be necessary for the development of a mesencephalic glutamatergic cell fate. Nakatani *et al*. (2007) showed in gain-of-function studies, that *Ngn1* promotes the glutamatergic phenotype. Overall our results demonstrate that *Uncx4.1* is required for the proper development of midbrain glutamatergic neurons.

## Conclusions

In this study, we have shown that the transcription factor Uncx4.1 is expressed in GABAergic, glutamatergic and dopaminergic neurons of the developing midbrain. A detailed analysis of the neuron population in the ventral midbrain of *Uncx4.1*^*−/−*^ embryos revealed a significant reduction in the content of mDA neurons. This study provides evidence that Uncx4.1 is a novel player in the complex regulatory network of mDA neuron differentiation and that it plays a role in the transition phase from progenitors to mature mDA neurons. Furthermore, our study uncovers that the loss of Uncx4.1 provokes an increase in Pax6-positive neurons in the ventral midbrain, suggesting another important function for Uncx4.1 in midbrain glutamatergic neurogenesis.

## Methods

### Animals

Animal experimentation and housing was performed in agreement with the regulations of the animal welfare law of Germany and approved by LAVES Institution of Low Saxony (Approval Nr.:33.9-°©‐‐4250204-°©‐‐11/0402).

Generation and genotyping of *Uncx4.1*^*−/−*^[12], *Uncx4.1*^*flox/flox*^[13], Cre-ER [34] Ngn2^Kigfp^[52] and *Pax6*^*−/−*^[53] mice has been described previously. *Ngn2*^*Kigfp/Kigfp*^ are referred as *Ngn2*^*−/−*^ in this article. Either wild-type animals or *Uncx4.1*-heterozygous animals were used as control for comparison with *Uncx4.1*^*−/−*^ embryos. Such controls are shown as *Uncx4.1*^*+/*?^. All mice were kept on B6N background.

For conditional ablation of *Uncx4.1* at E12.5 *Uncx4.1* heterozygous mice were first crossed with Cre-ER mice to obtain Cre-ER:*Uncx4.1*^*+/−*^ mice. These mice were further crossed to *Uncx4.1*^*fl/fl*^ mice. 1 mg/10 g body weight tamoxifen was injected to the pregnant female at E12.5. The morning of the vaginal plug was considered as E0.5.

### Immunohistochemistry, *in situ* hybridization and X-gal staining

Embryos were taken at the desired time point and used for *in situ* hybridization (ISH) or immunohistochemistry (ICH). Dissected brains or heads were placed in 4% PFA for 1 to 16 hours according to their size. After fixation, embryos were washed three times for ten minutes in 1 x PBS and, in case of cryoembedding followed by cryoprotection, in 30% sucrose (in 1 x PBS), 30 minutes in 50% tissue freezing medium (Jung; Leica, Nussloch, Germany) in 30% sucrose and embedded in tissue-freezing medium. In case of paraffin embedding, the tissue was dehydrated after washing in 1 x PBS through ascending ethanol series before it was transferred in isopropanol/toluene (through an ascending isopropanol/toluene series) and finally embedded in paraffin.

ISH using digoxigenin-labeled single-stranded RNA probes was performed on 18 μm thick cryosections according to Moorman *et al*. [54]. For two-colored ISH Dig- and Fluorescein-labeled probes were used. A detailed protocol for two-colored ISH is available upon request. The following *in situ* probes were used: *Ngn2*, *Helt*, *Mash1*, *Uncx4.1*, *Gad67*, *Lmx1a*, *Nkx2.2*, *Nkx6.1* and *Brn3a*.

For IHC, 10 μm thick cryo or paraffin sections were used. Paraffin sections were hydrated through descending ethanol series before boiling for one minute in unmasking solution (1:100 in water, Vector Laboratories, Burlingame, CA, USA). Afterwards, the sections were placed three times for five minutes in 1 x PBS prior to blocking (in 10% FCS + 0.01% Triton or 1% BSA + 0.01% Triton in PBS, sterile filtered). Primary antibodies were incubated overnight or for 72 hours at 4 °C in blocking solution. Secondary antibodies were diluted 1:750 in blocking solution and incubated for 70 minutes at room temperature. After secondary antibody sections were rinsed three times in 1 x PBS before mounting in Vectrashield with DAPI (Vector Laboratories). Cryosections were processed the same way without descending ethanol series and boiling.

Primary antibodies used were rabbit anti-Uncx4.1 (1:750), goat anti-Hnf3ß (1:150, Santa Cruz Biotechnology, Santa Cruz, CA, USA), rabbit anti-TH (1:300, Chemicon, Billerica, MA, USA), mouse anti-TH (1:5000, Sigma-Aldrich, St. Louis, MO, USA), rat anti-DAT (1:100, Santa Cruz Biotechnology), rabbit anti-Pitx3 (1:1000), goat anti-Nurr1 (1:100, R&D Systems, Minneapolis, MN, USA), rabbit anti-Pax6 (1:300, Covance, Princeton, NJ, USA), mouse anti-Pax6 (1:100, DSHB, Iowa City, IA, USA), rabbit anti-Nkx6.1 (1:3000 [55]), mouse anti-Nkx6.1 (1:100, DSHB), rabbit anti-Calbindin (1:200, Swant, Bellinzona, Switzerland), rabbit anti-Calretinin (1:300, Sigma-Aldrich), chicken anti-GFP (1:500, Abcam, Cambridge, MA, USA), anti-BrdU (1:50, Sigma-Aldrich), mouse anti-Ngn2 (1:100, R&D Systems) and rabbit anti-Olig2 (1:500, Chemicon). Secondary antibodies were either Alexa488- or Alexa594-conjugated and raised against mouse, rabbit, rat, chicken or goat (Invitrogen, Carlsbad, CA, USA).

X-gal staining was performed as describes previously [56].

Pictures were taken with an Olympus BX60 fluorescent microscope or with an Olympus SZX 12 fluorescent microscope (Olympus, Tokyo, Japan). Confocal pictures were taken with Leica TCS SP5 confocal microscope (Leica Microsystems, Germany). Pictures were processed with Adobe Photoshop (Version 10.0) by overlaying the pictures, adjusting brightness, contrast and size.

### BrdU labeling and TUNEL assay

100 μl/10 g bodyweight BrdU (15 mg/ml in PBS) was intraperitoneally injected into pregnant females at E10.5 or E14.5. In the case of the E10.5-day-old embryos, the animal was sacrificed 24 hours after BrdU injection. In the case of the E14.5-day-old embryos, the animal was sacrificed 45 minutes after the injection. Embryos were processed for embedding in cryo media, followed by IHC against anti-BrdU (1:100, Roche, Basil, Switzerland) or against mouse anti-BrdU (1:100, Roche) together with rabbit anti-TH (1:200, Chemicon). Apoptosis was detected using the TUNEL assay. The TUNEL Apoptosis detection kit (Millipore, Billerica, MA, USA) was used following the manufacturer’s instructions.

### Cell counting and statistical analysis

Cell counts were done on images of coronal sections or directly under the microscope along the rostral-caudal axis of the midbrain. For embryonic stages, every fourth and for adult stages every eighth section was counted and the average was calculated for at least three animals. The two-tailed unpaired Student’s *t* test was applied on the averages. Statistical significance was considered if *P* ≤0.05.

### Cell culture, Co-immunoprecipitation (Co-IP) and western blotting (WB)

Hela cells were cultured in DMEM medium with 10% fetal calf serum (FCS). For Co-IP the cells were seeded into a 10 cm dish. When 80 to 90% confluent cells were transfected using Lipofectamine 2000 (Invitrogen) following the manufacturer’s manual. During this process either DMEM or Opti-MEM serum-free medium was used. The next morning, the proteasome inhibitor MG132 (Sigma-Aldrich) was applied to the cells at a final concentration of 20 μm. Twenty-four hours after transfection the cells were harvested and Co-IP using FLAG tagged beads was performed according to the manufacturer’s recommendation (Sigma-Aldrich). For Co-IP Uncx4.1-c-myc was co-transfected with Ngn2-FLAG. Detailed information about the plasmids is available upon request. After performing the Co-IP the samples were applied on a 12% SDS gel. Afterwards, the proteins were transferred to a membrane overnight followed by 90 minutes blocking in 5% milk powder in PBT. After blocking, the first antibody was incubated overnight at 4 °C. The next day, the membrane was washed three times in 1 x PBT and the second antibody was applied for one hour at room temperature. After three times washing for 30 minutes in 1 x PBT the protein was detected using Super Signal West Pico and Super Signal West Femto kit (both Pierce, Rockford, IL, USA).

First antibodies used are rabbit anti-FLAG (1:1000 Sigma-Aldrich) and mouse anti-c-myc (1:500 Santa Cruz Technology). Secondary antibodies used are anti-rabbit-HRP (1:10000 Dianova, Hamburg, Germany) and anti-mouse-HRP (10000 Dianova).

## Abbreviations

BrdU: 5-bromo-2'-deoxyuridine; Co-IP: Co-immunoprecipitation; DA(T): Dopamine (transporter); E: Embryonic day; GFP: Green fluorescent protein; IHC: Immunohistochemistry; ISH: *in situ* hybridization; mDA: Midbrain dopaminergic neurons; Ngn2: Neurogenin 2; PBS: Phosphate buffered saline; RRF: Retrorubal field; SN(pc): Substantia nigra (pars compacta); TH: Tyrosine hydroxylase; TUNEL: Terminal deoxynucleotidyl transferase dUTP nick end labeling; VTA: Ventral tegmental area; VZ: Ventricular zone; WT: Wild-type.

## Competing interests

The authors declare that they have no competing interests.

## Authors’ contributions

TR designed the study, carried out most of the experiments and drafted the manuscript, SB produced the Uncx4.1 antibody, GG and BZ performed some of the ISH and IHC experiments, FV helped carry out the western blotting experiments, AK and ML provided the Uncx4.1 knockout ES cells for conditional KO as well as the Uncx4.1-c-myc tagged construct for protein expression, PB helped in conceiving the study and writing the manuscript. AM conceived the study, and participated in its design, coordination and helped to draft and write the manuscript. All authors read and approved the manuscript.

## Supplementary Material

Additional file 1 **Figure S1. ***Uncx4.1*-deficient midbrain shows no difference in cell proliferation at E11.5 as compared to control. **(A-B)** Immunohistochemistry (IHC) against 5-bromo-2'-deoxyuridine (BrdU) on coronal sections of E11.5 embryos after 24 hours of BrdU injection. **(C-D)** IHC against BrdU and tyrosine hydroxylase (TH) 24 hours after BrdU injection. BrdU was injected at E10.5 and the animals were sacrificed 24 hours later at E11.5. E, embryonic day. Click here for file

Additional file 2 **Figure S2.** Co-immunoprecipitation (Co-IP) of Uncx4.1 and Ngn2 provide no evidence for an interaction between the two factors. Hela cells were co-transfected as indicated and the lysates were used for a Co-IP assay using Flag tagged beads. Western blot (WB) analysis was performed using anti-c-myc and anti-Flag antibody. Click here for file

Additional file 3 **Figure S3.** Conditional inactivation of Uncx4.1 at E12.5 results in defects in the substantia nigra (SN) as documented by alterations in dopamine transporter (DAT) expression **(A-H)**, and tyrosine hydroxylase (TH) expression **(I-P)** in adult animals. The white arrows point to the region of the SN, which is affected in the mutant animals. Click here for file
