# Supplementary figures and images for: The transcription factor Uncx4.1 acts in a short window of midbrain dopaminergic neuron differentiation
Source: Neural Dev. 2012 Dec 8;7:39. doi: 10.1186/1749-8104-7-39 (PMC3558320; doi:10.1186/1749-8104-7-39)

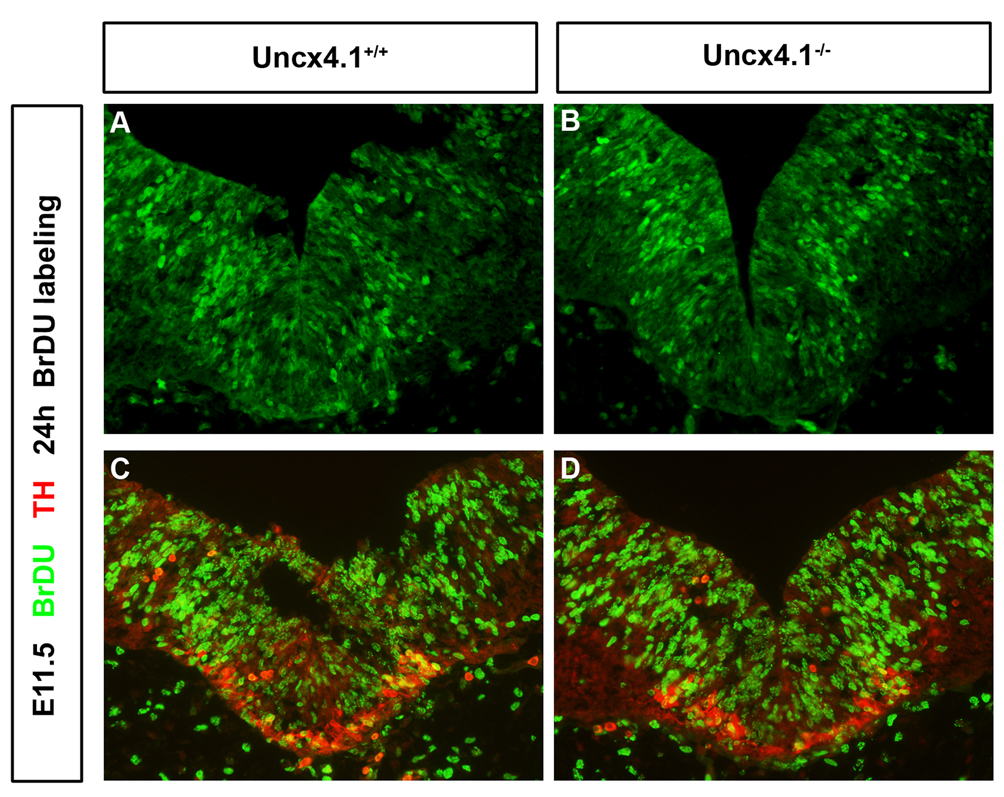

Supplement: Additional file 1 — Figure S1. Uncx4.1-deficient midbrain shows no difference in cell proliferation at E11.5 as compared to control. (A-B) Immunohistochemistry (IHC) against 5-bromo-2'-deoxyuridine (BrdU) on coronal sections of E11.5 embryos after 24 hours of BrdU injection. (C-D) IHC against BrdU and tyrosine hydroxylase (TH) 24 hours after BrdU injection. BrdU was injected at E10.5 and the animals were sacrificed 24 hours later at E11.5. E, embryonic day. [file 1749-8104-7-39-S1.jpeg]

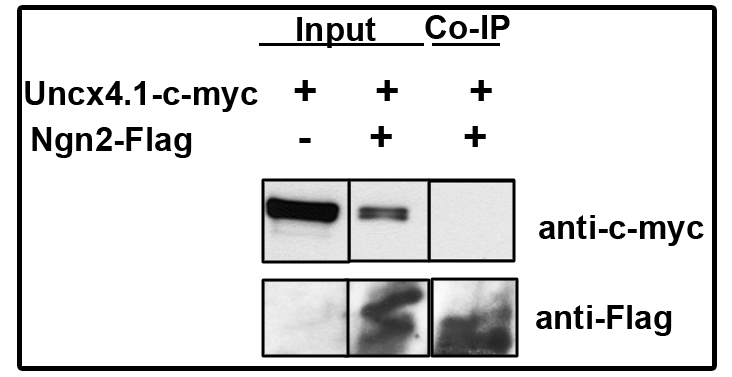

Supplement: Additional file 2 — Figure S2. Co-immunoprecipitation (Co-IP) of Uncx4.1 and Ngn2 provide no evidence for an interaction between the two factors. Hela cells were co-transfected as indicated and the lysates were used for a Co-IP assay using Flag tagged beads. Western blot (WB) analysis was performed using anti-c-myc and anti-Flag antibody. [file 1749-8104-7-39-S2.jpeg]

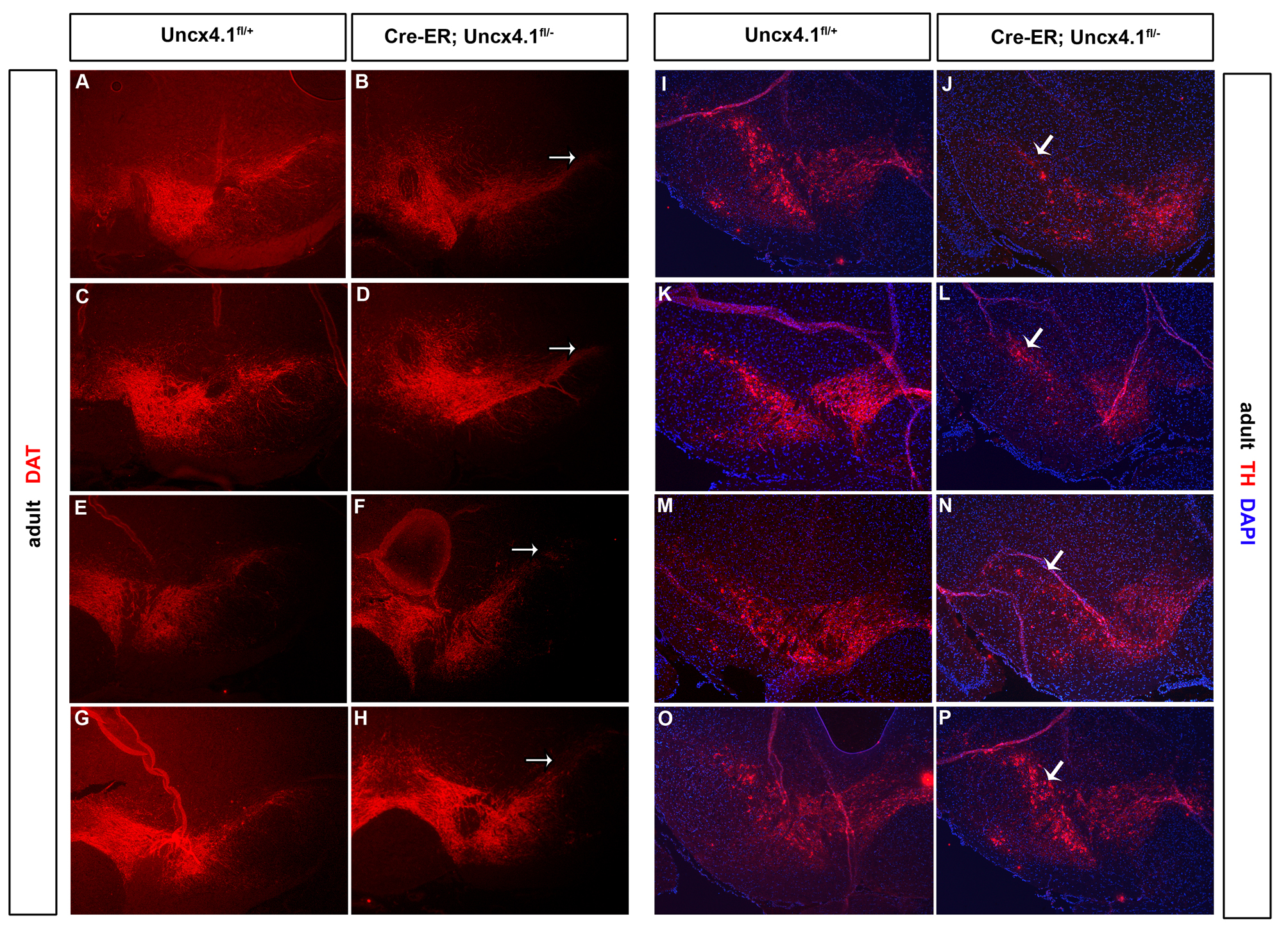

Supplement: Additional file 3 — Figure S3. Conditional inactivation of Uncx4.1 at E12.5 results in defects in the substantia nigra (SN) as documented by alterations in dopamine transporter (DAT) expression (A-H), and tyrosine hydroxylase (TH) expression (I-P) in adult animals. The white arrows point to the region of the SN, which is affected in the mutant animals. [file 1749-8104-7-39-S3.jpeg]
